# Low-calcium diet prevents fructose-induced hyperinsulinemia and ameliorates the response to glucose load in rats

**DOI:** 10.1186/s12986-015-0035-0

**Published:** 2015-10-29

**Authors:** Anna Voznesenskaya, Michael G. Tordoff

**Affiliations:** Monell Chemical Senses Center, 3500 Market Street, Philadelphia, PA 19104-3308 USA; Current address: The Rolf Luft Research Center for Diabetes and Endocrinology, Karolinska Institutet, Karolinska Hospital L1:02, Stockholm, SE 171 76 Sweden

**Keywords:** Calcium, Insulin resistance, Vitamin D, Glucose tolerance, Obesity

## Abstract

**Background:**

Consuming a fructose-rich diet leads to hyperinsulinemia, impaired glucose tolerance, and insulin resistance. In humans, the consumption of high levels of refined sugars often coincides with a diet containing suboptimal levels of calcium. Calcium and carbohydrate metabolism interact, so there is potential for fructose to have different health outcomes depending on whether the diet is calcium-rich or calcium-poor.

**Methods:**

We evaluated the metabolic effects of feeding fructose to rats that were maintained on either a calcium-replete diet or a low-calcium diet. Growing male Sprague Dawley rats were fed diets based on the AIN-93G formulation, with the main source of carbohydrate derived either from a mixture of cornstarch and sucrose or from fructose. Half the rats given each carbohydrate source were fed calcium at recommended levels (125 mmol/kg Ca^2+^); the others were fed a diet low in calcium (25 mmol/kg Ca^2+^). At various times, glucose and insulin tolerance tests were conducted to assess glucose metabolism.

**Results:**

Rats fed low-calcium diet had lower fasting insulin levels irrespective of the carbohydrate source they ate. They had a normal glycemic response to a glucose load and did not develop hyperinsulinemia under conditions of fructose feeding. The drop in blood glucose levels in response to insulin injection was larger in rats fed low-calcium diet than in those fed calcium-replete diet.

**Conclusions:**

Low-calcium diet prevented fructose-induced hyperinsulinemia and improved glucose handling under conditions of fructose feeding. Potential mechanisms underlying these effects of the low-calcium diet remain to be determined, but possibilities include impairment of insulin release from the pancreas and improved peripheral insulin sensitivity.

## Background

Diet has a major impact on metabolic health, and is a primary factor implicated in metabolic syndrome and Type 2 diabetes [1, 2]. The drive for energy-dense foods often leads to diets high in sugary drinks and snacks, resulting in high intakes of refined sugars and suboptimal intakes of micronutrients, such as calcium. The deleterious effects of excess consumption of simple sugars on carbohydrate metabolism are well recognized, with contributions made by both the quantity and the type of sugar consumed [2–4]. Due to a general increase in sweetener usage during the last decades of the 20^th^ century, and the popularity of high-fructose corn syrups, fructose consumption in the USA has risen by over 25 % [5, 6], although intakes may have decreased slightly in the last few years [7].

Fructose is a simple sugar contained in many food items, either by itself or as a part of the sucrose molecule. Diets rich in fructose induce impaired glucose tolerance accompanied by insulin resistance and hyperinsulinemia [8]. The mechanism underlying fructose-induced metabolic syndrome is complex. It is most likely initiated when fructose induces sustained hepatic gluconeogenesis and lipogenesis, because it bypasses the major regulatory steps of the glycolysis pathway [8, 9]. The action of fructose in the liver is thought to also induce insulin resistance in the periphery, followed by compensatory hyperinsulinemia [6, 10].

The progressive increase in intake of high-fructose corn syrup is related to the rise in the consumption of soft drinks, for which it is the main sweetener (e.g., [11]). One other major consequence of the increased intake of sweetened soft drinks is the displacement of milk from the diet [12]. Milk is an important source of calcium, so its disappearance from the diet has exacerbated suboptimal calcium intakes [12, 13]. Calcium deficiency leads to impaired insulin release in experimental animals, and there are indications that the same is true for humans [14–16]; decreases in circulating levels of both calcium and vitamin D are associated with changes in glucose homeostasis [17–19]. Thus, calcium and sugar intake, and their homeostasis are intertwined; however, little is known about how calcium status influences the regulation of metabolism under conditions of sugar overconsumption.

We investigated whether the combination of a high-fructose diet along with marginally inadequate calcium might have combined detrimental influences on glucose metabolism. To this end, we compared the metabolic profiles of rats fed diets with the carbohydrate provided either as a standard mixture of cornstarch and sucrose or as fructose, and with the calcium provided either at replete or marginally deficient levels. We found that relative to calcium-replete controls, rats fed low-calcium diet had improved insulin sensitivity and did not develop hyperinsulinemia when fed the high-fructose diet.

## Methods

The experiment involved a 2 × 2 design: Rats were fed diets containing either 125 or 25 mmol/kg Ca^2+^, with the carbohydrate source derived from either a standard sucrose-cornstarch mixture or from fructose. Blood samples were collected at various times, as described below.

The protocol was approved by the Monell Chemical Senses Center Institutional Animal Care and Use Committee.

### Subjects

Two identical cohorts of 36 rats each (72 total) were tested. Male Sprague–Dawley rats were obtained from Charles River (Raleigh, NC) at the age of 21 days. They were housed individually in stainless steel wire mesh hanging cages (19.5 × 17.5 × 24 cm). Unless otherwise noted, pelleted AIN-93G diet modified to contain 125 mmol Ca^2+^/kg (Table 1) and deionized water were provided ad libitum. The vivarium was maintained at 23 °C on a 12:12 h light/dark cycle with lights off at 19:00 and on at 07:00.

**Table 1:** Composition of experimental diets

		AIN125		AIN25		FRU125		FRU25	
Ingredient	kcal/g	g/kg	kcal/kg	g/kg	kcal/kg	g/kg	kcal/kg	g/kg	kcal/kg
Casein	3.58	200	716	200	716	200	716	200	716
L-Cystine	4	3	12	3	12	3	12	3	12
Fructose	3.8	0	0	0	0	617	2345	627	2383
Sucrose	4	100	400	100	400	0	0	0	0
Cornstarch	3.6	385	1386	395	1422	0	0	0	0
Dyetrose	3.8	132	501.6	132	501.6	0	0	0	0
Soybean oil	9	70	630	70	630	70	630	70	630
t-Butylhydroquinone	0	0.014	0	0.014	0	0.014	0	0.014	0
Cellulose	0	50	0	50	0	50	0	50	0
Mineral Mix #213019 (w/o Ca)	0.88	35	30.8	35	30.8	35	30.8	35	30.8
Vitamin Mix # 310025	3.87	10	38.7	10	38.7	10	38.7	10	38.7
Choline bitartrate	0	2.5	0	2.5	0	2.5	0	2.5	0
Calcium carbonate	0	12.5	0	2.5	0	12.5	0	2.5	0
Total kcal per kg			3715		3751		3772		3810

All diets were purchased from Dyets Inc, Bethlehem, PA (catalogue nos: 103660, 103662, 103661, 103663, respectively). Dyetrose is a proprietary dextrinized cornstarch used to facilitate pelleting the diet. The mineral and vitamin mixes yield energy because they are mixed in a powdered sucrose base

### Diets

The composition of the four experimental diets is shown in Table 1. AIN-93G diet is a diet recommended by the American Institute of Nutrition (now the American Society for Nutrition) in 1993 for growing rats. It contains approximately 10 % sucrose and 52 % cornstarch by weight (Table 1). Fructose-based diets were identical to the AIN-93G-based diets except that virtually all the carbohydrate was provided as fructose (small amounts of sucrose were used as a vehicle for the mineral and salt mixes). These were provided in either “replete” (125 mmol/kg) or “low” (25 mmol/kg) calcium concentrations. The 125 mmol/kg calcium concentration is well above requirements for growth [20, 21]. The 25 mmol/kg calcium concentration was chosen based on our previous work showing that it effectively reduced blood calcium concentrations and induced the motivation to consume calcium but it did not cause a failure to thrive (e.g., [22, 23]). For convenience, we abbreviate the diets based on their carbohydrate source [i.e., similar to AIN-93G or fructose (FRU)] and the concentration of calcium (125 or 25 mmol/kg).

### Procedures

After 3 days to acclimatize to the vivarium environment, the rats were assigned to four groups matched for body weight and the percentage of body weight that was fat, which was assessed using a Bruker Minispec nuclear magnetic resonance analyzer (see below). The experimental diets were introduced in two steps: On Day 0 (at age 24 days), half of the rats were switched to AIN25 diet; the other half remained on AIN125 diet. On Day 3, half of the rats in each AIN125 and AIN25 diet group were switched to fructose-based diets (FRU125 and FRU25). Thus, there were 18 rats in each of the four groups.

The body weight, food intake, and water intake of each rat was recorded daily. Body weight (±0.1 g) was measured using a top-loading balance. Food intake (±0.1 g) was based on the difference in weight between food given each day and food remaining the following day; it was corrected for spillage, which was collected under each cage. Body composition was assessed weekly. To accomplish this, each rat was carried to an adjacent room housing a Bruker Minispec LF110, placed into a plastic restraining tube, and then inserted into the core of the machine for ~90 sec while its body composition was assessed by magnetic resonance technology. The rat was then returned to its home cage.

To confirm the development of calcium deficiency, blood ionized calcium levels were measured on Days 10 and 17, at ~7 h after the start of the light period. Approximately 70 μl of blood was collected from the tip of the tail into a heparin-coated capillary tube (RAPIDLyte Multicap, Siemens) and within 15 sec analyzed using a Rapidlab 348 blood gas analyzer (Siemens, Germany) located in the vivarium. For this and subsequent tests, care was taken to collect blood samples from the rats in an order that controlled for disturbances and any delays between sampling and assaying. To do this, the samples were collected successively from rats in tetrads containing one representative of each of the four diet groups, and the order within each tetrad was counterbalanced.

Fasting blood glucose, insulin and triglyceride (TG) levels were measured in blood samples collected on Days 11, 18 and 42, at ~7 h after the start of the light period. Taking into account that young growing rats were relatively small at the beginning of the experiment we opted to use a morning fast instead of more severe overnight food deprivation: In small rodents prolonged overnight fasting induces a catabolic state, and along with standard housing conditions at subthermoneutral temperatures it can decrease metabolic rate [24]. In our experiment, we aimed to collect measurements under less extreme physiological conditions. Rats were fasted for 5–6 h and then a blood sample was collected from the tip of the tail into an EDTA-treated tube. The tubes were centrifuged and plasma glucose and TG levels were analyzed using enzyme-based colorimetric assays (Cayman Chemical, MI). Insulin levels were measured using ELISA (Rat insulin kit # EZRMI-13 K, EMD Millipore, MA). Sometimes when the rats were young, insufficient blood was collected to conduct all the measurements. In these cases, insulin measurements were given priority.

An oral glucose tolerance test was given between Days 23–25, at ~7 h after the start of the light period. After an overnight fast, each rat was gavaged with 8 ml/kg of 25 % glucose solution (2 g/kg). Blood was collected at 0, 15, 30, 60 and 120 min. Plasma glucose and insulin levels were measured as described above.

An insulin tolerance test (ITT) was given on either Day 36 or 37, at ~7 h after the start of the light period. After a 5–6 h fast, each rat received insulin [0.75 IU/kg, 0.75 ml/kg, i.p. of 1 IU insulin (HumilinR, Eli Lilly) solution prepared in isotonic saline]. For this test, glucose levels were measured using an Accu-Check Aviva Plus blood glucose meter (Roche Diagnostics).

### Data analysis

Body weights were analyzed using weekly measurements taken on the day of the body composition assessment. Daily food intakes were averaged into weekly blocks, based on the sum of daily measurements divided by the number of days; days when rats were fasted overnight were excluded from analysis.

Areas under the glucose and insulin curves during oral glucose tolerance tests were calculated using the trapezoidal rule [25, 26].

Two-way ANOVAs with factors of Diet Carbohydrate and Diet Calcium were used to analyze the area under the glucose and insulin curves. For all other measures, three-way mixed-design ANOVAs were used, with the same two between-subject factors and Time as a within-subject factor. When appropriate, differences between pairs of means were assessed using Fisher LSD tests. The criterion for statistical significance was *p* ≤ 0.05.

One animal was excluded from the analyses of the oral glucose tolerance test due to unsuccessful gavage of the glucose load. The Week 3 body composition of one rat was lost due to a technical error.

## Results

### Body weight and food intake

**Fig. 1:**
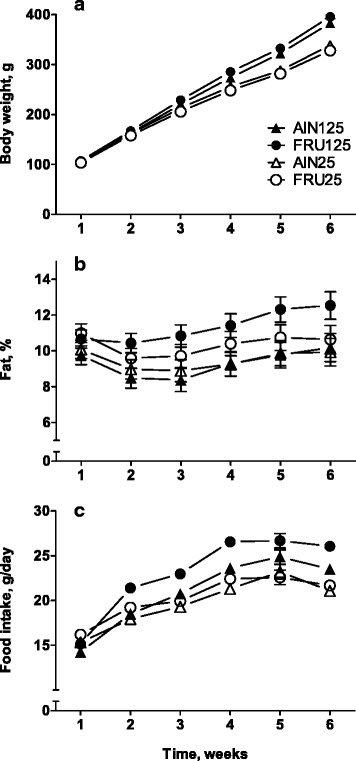
Body weight (**a**), body fat (as a proportion of body weight) (**b**), and food intake (**c**) of rats fed diets differing in carbohydrate source and calcium content. Symbols with vertical bars represent means ± SEMs. AIN125–rats fed AIN-93G diet containing 125 mmol Ca^2+^/kg, FRU125–rats fed fructose-based diet containing 125 mmol Ca^2+^/kg, AIN25–rats fed AIN-93G diet containing 25 mmol Ca^2+^/kg, FRU25–rats fed fructose-based diet containing 25 mmol Ca^2+^/kg. Where error bars are not shown they were smaller than the symbol size

All four groups of rats gained weight steadily over the 6 weeks of the experiment, although those fed the low-calcium diets gained less weight than did those fed the calcium-replete diets; the difference was statistically significant at Week 3 and thereafter [Diet Calcium × Time, F (5,335) = 99.6, *p* < 0.001]. The source of dietary carbohydrate did not affect body weight (Fig. 1a). Dietary calcium content had an effect on fat accumulation over time (Diet Calcium × Time, F (5,330) = 4.1, *p* ≤ 0.05; Fig. 1b): In all four groups, the proportion of body fat fell from Week 1 to Week 2, but in rats fed the calcium-replete diets the proportion of body fat increased significantly between Week 2 and Week 4, whereas in the rats receiving low-calcium diets a significant increase above the values for Week 2 did not occur until Week 5 (Fig. 1b). However, there were no significant differences between rats fed calcium-replete and low-calcium diet in the proportion of body fat at any time.

Food intakes reflected the differences in body weight gain: Starting from Week 3, rats fed the low-calcium diets ate significantly less food than did rats fed the calcium-replete diets (Diet Calcium × Time, F (5,335) = 17.9, *p* < 0.001; Fig. 1c). Fructose affected food consumption on Weeks 2 and 4 only, when the rats fed the fructose-based diet ate more food than did the rats fed the AIN diets [Diet Carbohydrate × Time, F (5,335) = 2.36, *p* ≤ 0.05].

### Fasting blood calcium, insulin, glucose, and TGs

Rats fed the low-calcium diets were hypocalcemic on Days 10 and 17, F (1,67) = 17.3, *p* < 0.001, with no difference between the two measurements, suggesting that the diets had their intended effects, and that blood ionized calcium concentrations were stable. There was no effect of the carbohydrate source on blood ionized calcium concentrations (Table 2).

**Table 2:** Fasting plasma levels of calcium, insulin, glucose and triglycerides (TG) on various days after introduction of experimental diets

	AIN125	FRU125	AIN25	FRU25
Ca^2+^, mg/dL^***^				
Day 10	5.60 ± 0.08	5.68 ± 0.08	5.36 ± 0.08	5.28 ± 0.04
Day 17	5.52 ± 0.08	5.60 ± 0.08	5.28 ± 0.08	5.28 ± 0.08
Insulin, ng/mL^**§^				
Day 11	2.0 ± 0.3	2.6 ± 0.3	1.9 ± 0.3	1.9 ± 0.3
Day 18	2.4 ± 0.3	3.3 ± 0.3	2.2 ± 0.3	2.3 ± 0.3
Day 42	4.7 ± 0.5	5.8 ± 0.4	4.0 ± 0.5	4.4 ± 0.5
Glucose, mg/dL^#§^				
Day 11	101 ± 4	109 ± 4	97 ± 4	105 ± 4
Day 18	115 ± 1	126 ± 4	116 ± 4	123 ± 4
Day 42	134 ± 3	143 ± 3	129 ± 3	138 ± 3
TG, mg/dL^#§^				
Day 11	56 ± 15	134 ± 16	48 ± 15	102 ± 14
Day 18	82 ± 16	173 ± 17	61 ± 16	163 ± 15
Day 42	98 ± 18	201 ± 19	75 ± 18	153 ± 16

Data are presented as mean ± SEM of *n* = 14–18 per group. Blood samples were collected during the light period, after a 5–6 h fast except for the calcium measurements when rats were in a fed state. ** - main effect of Dietary Calcium *p* < 0.01; ***-main effect of Dietary Calcium *p* < 0.001; #-main effect of Diet Carbohydrate *p* < 0.001; §-main effect of Time *p* < 0.001, Day11 < Day18 < Day42

Rats fed the diets containing fructose had elevated fasting glucose levels and markedly elevated TG levels relative to rats fed the AIN-based diets [glucose, F (1,62) = 13.4, *p* < 0.001; TG, F (1,50) = 36.7, *p* < 0.001]. There were no effects of dietary calcium on fasting blood levels of glucose or TG at any time; however, fasting insulin levels were lower in rats fed the low-calcium diets than calcium-replete diets, F (1, 64) = 6.9, *p* < 0.01. Fructose-fed rats tended to have higher fasting insulin levels than did rats fed the AIN-based diets, F (1,64) = 3.8, *p* = 0.06. There was no interaction between dietary carbohydrate and calcium [Diet Carbohydrate × Diet Calcium, F (1,64) = 1.9, NS; Diet Carbohydrate × Diet Calcium × Time, F (2,128) = 0.1, NS]. All four groups had increasingly higher glucose, TG and insulin concentrations as the experiment progressed [effects of Time: glucose, F (2, 124) = 71.9; TG, F (2, 100) = 20.9; insulin F (2, 128) = 75.6 *p* < 0.001].

### Oral glucose tolerance test

**Fig. 2:**
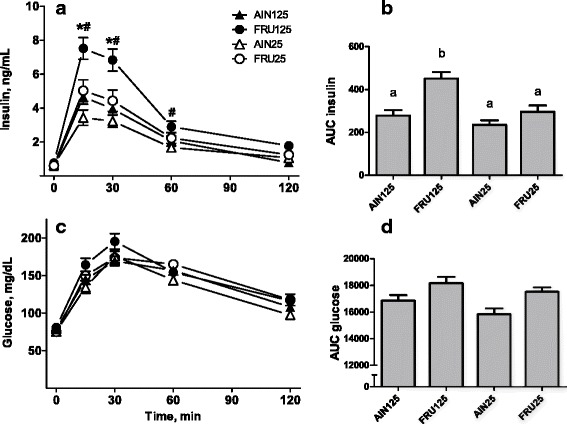
Oral glucose tolerance test of rats fed diets differing in carbohydrate source and calcium content. Symbols and bars show means ± SEMs. AUC–area under the curve, AIN125–rats fed AIN-93G diet containing 125 mmol Ca^2+^/kg, FRU125–rats fed fructose-based diet containing 125 mmol Ca^2+^/kg, AIN25–rats fed AIN-93G diet containing 25 mmol Ca^2+^/kg, FRU25–rats fed fructose-based diet containing 25 mmol Ca^2+^/kg; groups sharing the same letter did not differ from each other. Changes in plasma insulin levels (**a**): *Diet Calcium × Time, *p* < 0.001; # - Diet Carbohydrate × Time, *p* < 0.001. Insulin AUC (**b**): Diet Carbohydrate x Diet Calcium, *p* ≤ 0.05. Changes in plasma glucose levels (**c**): 0 no differences; 15: AIN125^a^, FRU125^b^, AIN25^a^, FRU25^ab^; 30: AIN125^a^, FRU125^b^, AIN25^a^, FRU25^a^; 60: AIN125^a^, FRU125^ab^, AIN25b^ab^, FRU25^abc^; 120: AIN125^ab^, FRU125^ab^, AIN25^a^, FRU25^ab^; Glucose AUC (**d**): There were no differences between individual means. Diet Carbohydrate (*p* < 0.001) and Diet Calcium (*p* ≤ 0.05) independently affected the glucose AUC

Dietary fructose and calcium had independent and opposite effects on the elevation of plasma insulin concentrations elicited by the oral glucose load: Fructose feeding increased insulin concentrations whereas the low-calcium diet decreased them (Fig. 2a; Diet Carbohydrate × Time, F (4,240) = 10.2, *p* < 0.001; Diet Calcium × Time, F (4,240) = 8.1, *p* < 0.001). Calcium-replete rats fed fructose had an exaggerated insulin response compared with the other three groups (Fig. 2b, AUC: Diet Carbohydrate × Diet Calcium, F (1,67) = 4.3, *p* ≤ 0.05).

The effect of fructose consumption on plasma glucose concentrations depended on calcium status (Fig. 2c, Diet Carbohydrate × Diet Calcium × Time, F (4,236) = 2.5, *p* ≤ 0.05). The glucose levels of the FRU125 group were higher than those of the other three groups at 30 min after gavage, and higher than those of the AIN125 and AIN25 groups, but not the FRU25 group, at 15 min after gavage. The plasma glucose levels of rats fed the FRU25 diet were similar to those of rats in the other three groups at every time. The area under the curve was smaller in rats fed the low-calcium diets than the replete diets, F (1,67) = 3.89, *p* ≤ 0.05 and was higher in the fructose-fed rats than in the rats fed AIN-based diets F (1,67) = 12.6, *p* < 0.001 (Fig. 2d).

### Insulin tolerance test (ITT)

**Fig. 3:**
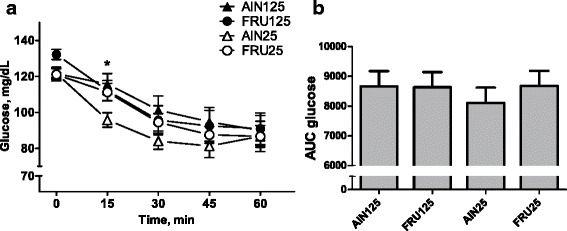
Insulin tolerance test. Data are means ± SEMs. AUC–area under the curve, AIN125-rats fed AIN-93G diet containing 125 mmol Ca^2+^/kg, FRU125–rats fed fructose-based diet containing 125 mmol Ca^2+^/kg, AIN25–rats fed AIN-93G diet containing 25 mmol Ca^2+^/kg, FRU25 – rats fed fructose-based diet containing 25 mmol Ca^2+^/kg; (**a**) Changes in plasma glucose levels: *-AIN125^b^, AIN25^a^, FRU125^ab^, FRU25^ab^. Glucose AUC (**b**): No significant differences

Rats fed the low-calcium diets had lower plasma glucose concentrations in response to insulin administration, but this depended on the carbohydrate source (Fig. 3; Diet Carbohydrate × Diet Calcium × Time, F (5,340) = 2.2, *p* ≤ 0.05). Glucose levels at 15 min after insulin injection were lower in the rats fed AIN25 diet than in those fed AIN125. There were no other differences.

## Discussion

The results demonstrate that fructose-induced hyperinsulinemia and impaired glucose tolerance depend on calcium status: In line with earlier work, plasma insulin concentrations and blood glucose levels in response to a glucose load were markedly elevated in calcium-replete rats fed fructose relative to those fed a standard starch-and-sucrose mixture [10]. These effects of fructose feeding were strongly attenuated in rats that received a low-calcium diet. However, fasting glucose levels in fructose-fed rats were not improved by low-calcium feeding. Thus, eating a low-calcium diet significantly alleviated but did not abolish the effects of fructose feeding on glucose handling.

Relative to the calcium-replete fructose-fed rats, the calcium-deficient fructose-fed rats had lower glycemia in response to a glucose load despite a smaller rise in insulin levels. This implies that the response to insulin is less affected by fructose overfeeding under conditions of calcium deficiency. The drop in blood glucose levels in response to insulin injection was larger in rats that received the low-calcium version than calcium-replete version of the AIN-93G diet. This points towards increased insulin sensitivity of the peripheral tissues under conditions of calcium deficiency.

There are at least two plausible mechanisms by which calcium deficiency could influence insulin sensitivity. One involves the activation of insulin receptor expression by high levels of circulating 1,25-hydroxyvitamin D_3_ [27–29]. An increase in levels of 1,25-hydroxyvitamin D_3_ is a classic effect of calcium deficiency, and it has been observed with the low-calcium diet used here (i.e., AIN25 diet; [30, 31]). Activation of insulin receptor expression through 1,25-hydroxyvitamin D_3_ action would counteract the suppression of insulin receptor responsiveness that is exerted by fructose feeding [32]. The other plausible mechanism involves activation of the osteoblast-specific factor, osteocalcin. Levels of circulating osteocalcin, including the active uncarboxylated form, increase when rats are fed low-calcium diets [33, 34]. This improves insulin sensitivity in adipose tissue and liver [35]. Consistent with this, injections of osteocalcin improve insulin sensitivity in mice fed high-fat diet, which is a metabolic challenge comparable in some of its effects to fructose feeding [36].

Enhanced insulin sensitivity mediated through the action 1,25-hydroxyvitamin D_3_ and/or osteocalcin may have emerged as an adaptation to conditions when dietary sources of calcium are scarce in order to overcome the secretory deficiency of pancreatic beta-cells induced by low calcium levels.

Reduced fasting insulin levels in rats fed low-calcium diets, and hampered glucose-induced hyperinsulinemia in fructose-fed rats receiving low-calcium diets, is consistent with the effect of dietary calcium deficiency to interfere with insulin release [14, 15]. Glucose-induced hyperinsulinemia in fructose feeding may have also been ameliorated by what appears to be improved insulin sensitivity in calcium deficiency: Exaggerated insulin release in fructose feeding is thought to be a compensatory response to the development of insulin resistance in the periphery and thus could potentially be prevented if insulin sensitivity is maintained [6].

Insulin sensitivity is tied to body size, particularly body fat. However, this cannot explain the results found here because differences in insulin release, and the effects of insulin on blood glucose levels, in rats fed the low-calcium and the calcium-replete diets were not related to the proportion of adipose tissue: There were no differences in the proportion of body fat among the experimental groups at any time.

Several studies suggest that, under some conditions, calcium-deficient rodents are more susceptible to obesity than their replete controls [37, 38] but there are also several failures to observe this [31, 39]. The current work falls in the latter camp and supports the conclusion that calcium intake has no direct effect on the development of obesity in rats.

## Conclusions

The experiments reported here reveal that, in rats, a low-calcium diet—despite being harmful in its own ways [40–43]—can counteract the detrimental effects of fructose overconsumption on glucose handling. If this relationship applies to humans, then exhortations to consume more calcium may exacerbate components of metabolic syndrome. The health outcomes caused by excess consumption of high-fructose corn syrup may be influenced as much by the calcium status of an individual as by the amount of the sugar the individual consumes. At the least, the results point to the complexity of nutritional interactions that make giving dietary advice fraught with peril.
